# Contextualization, Procedural Logic, and Active Construction: A Cognitive Scaffolding Model for Topic Sentiment Analysis in Game-Based Learning

**DOI:** 10.3390/bs15101327

**Published:** 2025-09-27

**Authors:** Liwei Ding, Hongfeng Zhang, Jinqiao Zhou, Bowen Chen

**Affiliations:** Faculty of Humanities and Social Sciences, Macao Polytechnic University, Macao, China; dinglw0405@gmail.com (L.D.); zhoujinqiao6@gmail.com (J.Z.); p2312561@mpu.edu.mo (B.C.)

**Keywords:** game-based learning, situated learning theory, LDA topic modeling, sentiment analysis, cognitive scaffolding

## Abstract

Following the significant disruption of traditional teaching by the COVID-19 pandemic, gamified education—an approach integrating technology and cognitive strategies—has gained widespread attention and use among educators and learners. This study explores how game-based learning, supported by situated learning theory and game design elements, can boost learner motivation and knowledge construction. Using 20,293 user comments from the Chinese video platform Bilibili, the study applies sentiment analysis and LDA to uncover users’ sentimental tendencies and cognitive themes. The analysis identifies four core themes: (1) The application of contextual strategies in language learning, (2) Autonomous exploration and active participation in gamified learning, (3) Progressive enhancement of logical thinking in gamified environments, and (4) Teaching innovation in promoting knowledge construction and deepening. Building on these findings, the study further develops a cognitive scaffolding model integrating “contextualization–procedural logic–active construction” to explain the mechanisms of motivation–cognition interaction in gamified learning. Methodologically, this study innovatively combines LDA topic modeling with sentiment analysis, offering a new approach for multidimensional measurement of learner attitudes in gamified education. Theoretically, it extends the application of situated learning theory to digital education, providing systematic support for instructional design and meaning-making. Findings enrich empirical research on gamified learning and offer practical insights for optimizing educational platforms and personalized learning support.

## 1. Introduction

Gamification refers to the application of game design elements and principles in non-game situations, and can also be understood as a series of activities and processes that deal with specific problems by applying or borrowing the characteristics of game elements (Behl et al., 2022). In recent years, the application and practice of gamification have attracted widespread attention, and this trend has attracted profound attention and discussion in various industries and academic fields, such as education (Khaitova, 2021; Oliveira et al., 2023), medical care (Damaševičius et al., 2023; M. S. Patel et al., 2021), social services (Ciuchita et al., 2023; Martinho et al., 2020). With the development of technology and the changes in educational needs, the potential and application prospects of gamified education are becoming more and more broad. Not only has it changed the traditional teaching methods, it also provides students with a more interactive and immersive learning environment.

Gamification has long penetrated education and has attracted widespread academic attention and research. Early educational practice focuses on introducing gamified elements into classroom teaching, which not only significantly promotes the practice and research of flipped classrooms (Stöhr et al., 2020), but also achieves significant results (Zainuddin et al., 2020a). Under the influence of the COVID-19 pandemic, with the rapid development of technology, the field of education is undergoing profound changes (Rincon-Flores & Santos-Guevara, 2021). In this context, the need for systematic research on educational games is becoming increasingly prominent. Its design, development and application have become important tools to improve learning effects and promote flexible education, providing important support for coping with this transformation. Among them, Immersive Learning uses VR and enhanced AR technology to enable students to learn in virtual or enhanced environments, enhancing interaction and experience (Beck et al., 2023; Fitrianto & Saif, 2024) and Self-Directed Learning, combined with gamified elements, can stimulate students’ motivation to learn and help them better master knowledge and improve their skills (Pacheco-Velazquez, 2020; Palaniappan & Noor, 2022). Furthermore, Task-based Learning, by setting specific tasks and challenges, students need to learn and apply knowledge by solving problems and completing tasks (Pingmuang & Koraneekij, 2022; Sangtip & Negrn, 2021).

Current literature has thoroughly demonstrated that gamified education can enhance students’ learning interest and engagement through game elements (Khaleel et al., 2020). On one hand, the interactivity, immediate feedback, and reward mechanisms of gamification can effectively improve students’ learning motivation (Hellín et al., 2023). On the other hand, it provides a more flexible and diverse space for personalized learning experiences (Marougkas et al., 2021). Studies also indicate that gamified education significantly enhances students’ problem-solving abilities and self-regulation through task mechanisms (Li et al., 2022). For example, through competitive and interactive modes in task mechanisms, students are encouraged to leverage their strengths, enhancing their social skills and teamwork in collaboration, thereby achieving knowledge sharing and sentimental resonance (Alkurdi, 2021). Furthermore, in challenging tasks, gamification stimulates students’ intrinsic motivation, promotes active thinking, and encourages the exploration of various problem-solving strategies, effectively improving their self-monitoring and sentimental regulation, while strengthening their ability to manage the learning process (C. Chen et al., 2024).

With the increasing in-depth exploration of the impact of gamified education, researchers have begun to investigate how to more precisely analyze the sentimental changes of learners in dynamic learning environments. By using Aspect-based Sentiment Analysis (ABSA), the sentimens expressed in learners’ texts are linked to specific topics or aspects, thereby revealing the relationship between “learning content” and “sentimental responses” (X. Chen & Xie, 2020). In large-scale online learning environments, studies have shown that combining topic and sentiment modeling helps reveal the connections between learning behaviors and sentimental changes, thereby enhancing the accuracy of opinion tracking and intervention design. Furthermore, other research has found that learners’ sentimental expressions exhibit dynamic temporal characteristics, and that sentimental fluctuations and transitions are influenced by the learning phase (Liu et al., 2019). Therefore, combining time-series modeling with Aspect-based Sentiment Analysis can not only accurately characterize sentimental evolution patterns but also provide an effective analytical framework for sustained engagement and motivation changes in gamified education (Haslbeck et al., 2023).

Although a lot of research has been discussed in the implementation and effects of gamified education, there is still a lack of systematic research on how it shapes learners’ knowledge construction through game situations and game design elements. To fill this research gap, this paper introduces Situated Learning Theory as the core analytical framework (Cobb & Bowers, 1999), which believes that learning is closely related to real situations and emphasizes the profound impact of situations on the learning process. From this theoretical perspective, external environmental factors such as platform design, participation mechanism, task challenges, etc. are closely intertwined with individual cognitive activities (Aryulina, 2020), jointly promoting knowledge construction. Task-based situations and reward mechanisms in gamified elements can enable learners to experience contextualized learning during participation, thereby more effectively promoting independent exploration and active participation (Esteban, 2024).

In order to more in-depth analysis of learners’ sentimental attitudes and cognitive processes in gamified education situations, this study combines LDA models and sentiment analysis, and develops topic modeling and sentiment analysis based on user comments on gamified education-related software in social media. This approach is not only intended to reveal the mechanisms of the formation of learners’ sentimental attitudes, but also to explore how learners can construct knowledge through interactive and immersive experiences in this situation. Overall, this study hopes to deeply explore the interweaving process of the internal motivation and cognitive construction of learners’ sentimental attitudes in gamified education, and provide new perspectives and inspirations for educational theory and practice.

## 2. Research Design and Methodology

Sentiment analysis, also known as opinion mining, uses natural language processing (NLP) technology to detect and understand public sentimental responses to a particular product or service, as well as individuals or groups’ potential intentions in a specific situation (Mehta & Pandya, 2020; Nandwani & Verma, 2021). As one of the core technologies in the development of artificial intelligence (AI), sentiment analysis is widely used in corporate, government and academic research, helping relevant subjects extract and analyze public sentiments and opinions, and has great potential for wide application in various fields (Nandwani & Verma, 2021). In recent years, scholars have conducted in-depth research on sentiment analysis. For example, recurrent neural networks (RNN) and Word2vec algorithms are used to analyze sentiments written in Indonesia (Kurniasari & Setyanto, 2020), and research has also conducted sentiment analysis of customer review data based on Bi-GRU and Bi-LSTM architectures (Sachin et al., 2020), and scholars have used RNN and CNN to build a fusion model (Usama et al., 2020). In this study, in order to analyze the sentimental attitudes and theme sentimens expressed by the public in the context of “gamified education”, SnowNLP, an sentiment analysis tool based on the Chinese corpus, was used to conduct sentimental judgments, which further revealed the public’s sentimental attitudes to different educational themes, providing an important reference for a deep understanding of user feedback. In order to further reveal the semantic structure and theme attributes of text content, this paper also introduces theme modeling technology to deeply explore the text.

**Figure 1:**
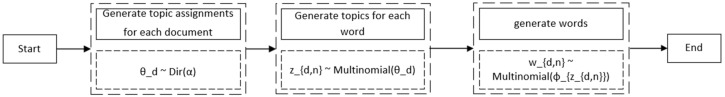
LDA topic extraction process.

LDA (Latent Dirichlet Allocation) is a topic modeling method (Chauhan & Shah, 2021) used to automatically identify potential topic structures from text corpus. The core idea of LDA is to use document sets as a probability distribution made up of multiple potential topics (Luong et al., 2024). As shown in Figure 1 of the LDA generation process, the model assumes that the distribution of each document on different topics is generated by the LDA distribution, and the vocabulary distribution under each topic also obeys the LDA prior distribution (Curiskis et al., 2020). In the text generation process, a topic distribution is first extracted for each document, and then for each word in the document, a topic is randomly selected according to the topic distribution of the document, and a specific vocabulary is sampled from the word distribution of the topic (Gan & Qi, 2021). This generation process reflects the subject structure of the document content, allowing LDA to automatically mine potential topic information from large-scale text data and quantify the weight of each document on different topics.

After using the LDA model to identify potential theme structures in the text, this study further focused on the sentimental distribution characteristics under different themes to comprehensively evaluate the user’s sentimental attitudes in each theme dimension. To achieve this goal, two complementary sentiment analysis methods were designed and implemented. The first method is based on the complete comment text, first conducts sentiment analysis on all comments, and then combines the topic-word distribution output by the LDA model to calculate the sentimental scores of each comment on each topic, thereby revealing its sentimental tendencies and differences under different topics. The second method adopts a keyword-driven theme sentimen mapping mechanism, calculates the similarity between the comment text and the theme keyword set extracted by LDA, classifies the comments to the most relevant topics, and then conducts sentimental analysis of the comment sets under each topic to form a more semantic and targeted topic sentimen distribution. The two methods achieve the integration of sentiment analysis and theme modeling from different dimensions, which not only improves the sentimental explanatory power of theme recognition results, but also provides a more solid empirical basis for public attitude insight and related strategy formulation.

**Figure 2:**
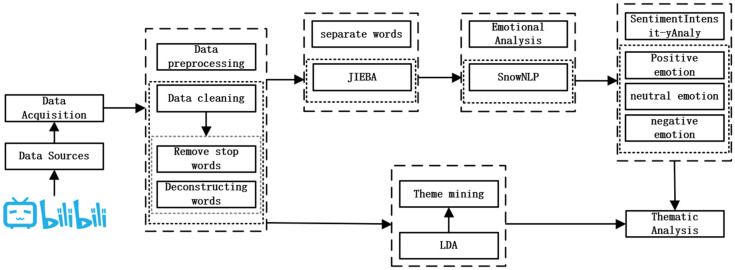
General diagram of research methods.

The process for this study is shown in Figure 2. First, Bilibili-related video comments were obtained, and deduplication, denoising and pre-processing were performed through manual and machine-combining methods (such as removing stop words and JIEBA participle). Then, sentimen analysis was performed in combination with the sentimen dictionary and SnowNLP scores, and sentimental tendency classification was performed on the comments. Finally, the LDA model is used to perform topic modeling, identify potential topic structures, and conduct multi-level topic sentiment analysis to reveal the sentimental characteristics and topic distribution of comments.

## 3. Methodology

### 3.1. Platform Selection and Data Processing

This study takes users of gamified education software as the research object, aiming to explore the user’s sentimental attitudes and user experience by analyzing their comment data after use. As the main data acquisition platform, Bilibili is significantly representative and reasonable. Official data shows that Bilibili has an average daily active user base of 107 million and an average monthly active user base of 348 million, indicating its high stickiness and wide coverage in China’s mainstream video and social platforms. It has outstanding performance in content fields such as games, knowledge sharing, and technology. It has rich user comments, barrage and feedback, which can reflect the user experience more realistically. Text data such as video comments, barrage, submission feedback and other related textual data on the Bilibili platform that are related to gaming education software can more realistically reflect the user’s actual user experience and sentimental reactions, and help improve the effectiveness and scientificity of research conclusions.

The data collection of this study follows the principle of real user feelings. In order to ensure that the data has high representativeness and accuracy, multiple rounds of screening were conducted, and comments that were not related to the research topic were eliminated. In Bilibili, relevant data collection was collected based on keywords such as “gamified education”, “gamified learning”, and “gamified software”, and a total of 38,112 user comment data. After preliminary collection, the data was deduplicated, noise reduction, and non-Chinese text. The data was cleaned up using Excel tables, removing null values, duplicates and irrelevant information, and finally 20,293 valid data were retained. Then, the data is preprocessed, and a program was written in python to remove stop words and participles. Simply put, it is to use the JIEBA word segmentation tool to remove irrelevant words and participles (including common persons, pronouns, preposition numbers and symbols, etc.).

### 3.2. Sentiment Analysis

A total of 20,293 comment data were collected from the Bilibili platform. Due to the large volume of data, sentiment analysis was performed using programming methods. The SnowNLP library was utilized for Chinese text sentiment analysis, and sentiment evaluation of a large number of texts was conducted through reading Excel files in bulk. First, the pandas library was used to load the input Excel file, and a SentimentAnalysis class was defined to perform sentiment analysis. This class contains two core methods: get_sentiment_score, which calculates the sentiment score of the text, and get_sentiment_label, which assigns a sentiment label (Positive, Negative, Neutral) to the text based on the sentiment score. Both the sentiment score and label division criteria rely on the Chinese sentiment analysis model built into the SnowNLP toolkit, which is trained on Chinese corpora and is more effective in reflecting the true sentimental tendencies of Chinese text, offering strong linguistic adaptability and practicality.

In this study, the tool employed is SnowNLP, a system specifically designed for Chinese text processing that can perform a range of natural language processing tasks without relying on deep learning frameworks. Compared with other methods and models—for instance, BERT, which demonstrates superior performance in terms of accuracy—SnowNLP does not show a clear disadvantage in Chinese sentiment analysis tasks. On the contrary, the complexity of the BERT model often introduces additional noise and computational redundancy, leading to less satisfactory performance in certain scenarios. Therefore, SnowNLP’s simplicity and efficiency make it more suitable for handling sentiment analysis tasks in Chinese. To evaluate the performance of SnowNLP, this study adopted a data extraction approach: 200 data entries were randomly sampled each time, repeated 5 times, for a total of 1000 samples used for testing. Expert-annotated labels were established as the ground truth (“expert labels”), while SnowNLP’s outputs were recorded as “Snow labels.” Using scikit-learn, we then calculated SnowNLP’s Accuracy, Precision, Recall, and F1 Score. As shown in Table 1, the results of the five tests indicate that the accuracy of each group remained stable between 0.71 and 0.75. Precision and recall ranged from 0.68–0.73 and 0.72–0.77, respectively, while the F1 scores clustered around 0.71–0.75, with only minor fluctuations. These results highlight the robustness and consistency of the model across different datasets. Overall, SnowNLP demonstrates relatively reliable performance in sentiment classification tasks, providing a feasible technical foundation for subsequent research and practical applications. Nevertheless, it should be noted that because this study employed random sampling from a large dataset for expert evaluation, discrepancies remain between human annotation and model predictions, which may influence the evaluation outcomes.

### 3.3. Topic Modeling

In LDA topic modeling, it is important to determine the optimal number of topics k (Gan & Qi, 2021). Setting the k value too small will result in the model being too simple to capture text diversity; setting it too large will result in duplicate and similar topics. To optimize the topic model and evaluate its effectiveness, this article determines the optimal number of topics by calculating coherence score. Specifically, the CoherenceModel class in the Gensim library and its c_v measurement method are used to calculate the consistency score to verify the rationality and interpretability of the extracted topic. During the experiment, the number of topics was set to 2 to 10, and the model effect of different topics was evaluated by consistency scores. The final experimental results show that the optimal number of topics is 4, and the consistency score fluctuates between 0.63 and 0.67 to achieve the best topic.

After determining that the number of topics is 4, the model showed good results in capturing the potential semantic structure of the data, providing solid support for in-depth understanding and representation of text topics. The experimental process uses the LdaMulticore function in the Gensim library to model the topic, and train the LDA model in parallel on multiple processors. Set the number of model training topics to 4 and perform 10 passes on the data to optimize the model’s inference process. At the same time, two worker threads are used to compute in parallel to accelerate the training process of the model. After the training is completed, the LDA model outputs the main keywords and their weights of each topic, which provide an important basis for in-depth understanding of the core content of each topic. Finally, domain experts were invited to validate the appropriateness of the selected number of topics from two dimensions: qualitative interpretability and topic uniqueness. Specifically, by manually analyzing the high-probability terms within each topic, the experts assessed their semantic coherence and domain consistency to ensure that each topic possesses clear semantic boundaries. This process helps guarantee that the topics effectively cover the core content of the research corpus while avoiding excessive overlap or ambiguity between topics. In addition, by independently running the LDA model multiple times and analyzing the results, it was found that when k = 4, the vocabulary within each topic was highly concentrated in specific semantic domains, with no overlap or cross-regional interference among the topics. This indicates that the model, at this topic number, exhibits a strong degree of topic uniqueness.

### 3.4. Topic Sentimen

In this study, in order to deeply explore the sentimental attitudes of users under different topics, a dual analysis strategy based on LDA topic modeling and sentiment analysis was adopted to model and measure the sentimental tendencies in text data from two dimensions.

**Figure 3:**
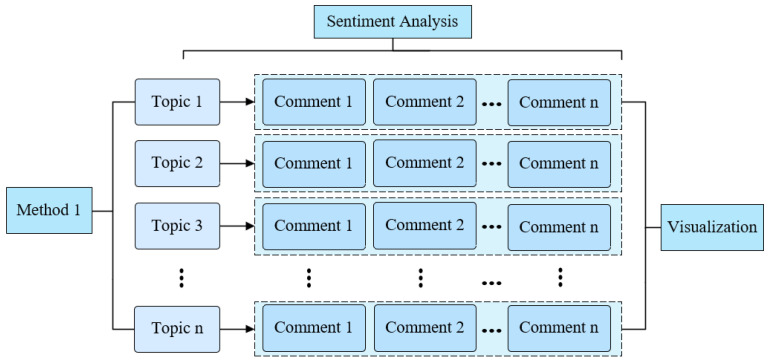
Method 1 Research Diagram.

Method 1: Sentimental fusion analysis based on the complete comment text. Taking the full review as the unit of analysis, SnowNLP is used to perform sentiment analysis on each review to obtain its overall sentimental score. Then, based on the topic-word distribution matrix output by the LDA model, the degree of correlation of the comment on each topic is calculated, and then combined with the sentiment scores to assign it to different topic dimensions, obtaining the weighted sentiment value under each topic. This method realizes the reverse mapping of sentiments from the overall sentimental feeling of each topic, helps to identify the sentimental differences in the same comment on multiple topics, and provides support for understanding the diverse sentimental attitudes contained in individual expressions. This multi-topic mapping approach helps capture the subtle differences and complexities of sentiments. The method enables reverse mapping from overall sentiment to sentiment for each individual topic, aiding in the identification of sentimental variations across different topics within the same review. As shown in Figure 3, the research framework for Method 1, this approach is suitable for analyzing users’ fine-grained sentimental attitudes in contexts where multiple topics are interwoven. However, when the review topics are more dispersed, a sentimental dilution effect may occur, resulting in less pronounced sentimental differences between topics. This, in turn, weakens the stability of the overall trend analysis.

**Figure 4:**
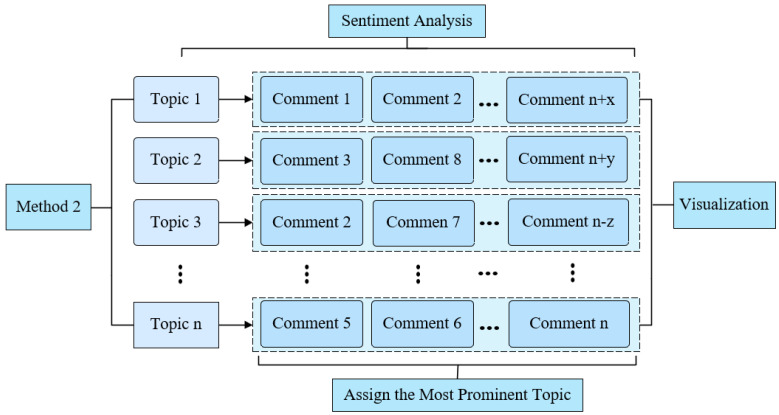
Method 2 Research Diagram.

Method 2: Topic sentiment mapping based on keyword similarity. Focus on constructing semantic connections between comment text and topic keywords. Specifically, firstly, the set of keywords generated by the LDA model is extracted, and the similarity between the comment text and the keyword set is calculated, and the comments are divided into the most relevant topic categories based on the principle of maximum similarity. Subsequently, the sentimental analysis was conducted on the comment collection classified under each topic to count the sentimental distribution characteristics under the topic. This method emphasizes the semantic clustering effect of the topic, which can effectively improve the pertinence and interpretability of the sentiment analysis results in the theme dimension. As shown in Figure 4, the research framework for Method 2, this approach is suitable for analyzing the overall trends in large-scale reviews. However, this aggregation method has certain limitations, namely that it may overlook secondary sentimental dimensions present in the reviews, and in complex contexts, it may fail to fully capture the multifaceted attitudes of the reviewers.

Overall, the first approach is suitable for in-depth analysis of individual comments’ sentiments, while the second approach is more suitable for topic classification and overall analysis of sentimental trends in large-scale data. Combining these two analytical methods can provide more comprehensive and accurate research results and conduct in-depth analysis based on the specific topics to which the review belongs, thereby drawing more accurate and multi-dimensional research conclusions.

## 4. Results

### 4.1. Sentimental Attitude

#### 4.1.1. Keyword Analysis

**Figure 5:**
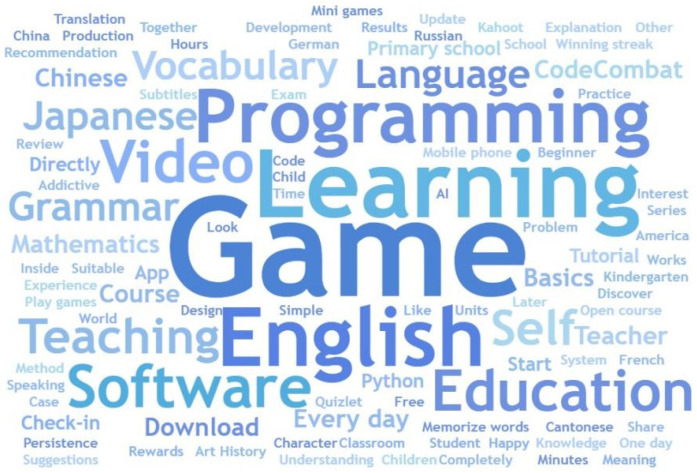
Keyword word cloud.

The dataset involves complex semantic analysis and diversified keywords. There are 20,561 keywords initially extracted, which belongs to the advanced dataset. Keywords with too low frequency will significantly increase the sparsity of the model. They contribute little to tasks such as classification and clustering, and may instead cause noise. Therefore, it is necessary to clear data with a frequency of less than or equal to three to ensure high validity of the data, and finally obtain 4166 effective keywords. The following table shows the top 10 keywords and their frequencies in descending frequency order. By analyzing these keywords and word cloud diagram Figure 5, we can see the recurrence of high-frequency words such as “game”, “learn”, “English”, “programme”, “video”, and “software”. This shows that discussions between language learning, program education and gamified education software are concentrated. These high-frequency words further emphasize the increasingly close relationship between learning and gamified educational tools, highlighting the importance of educational innovation, language learning, and programmatic thinking in contemporary education. (See Table 2).

#### 4.1.2. Sentimental Score Analysis

**Figure 6:**
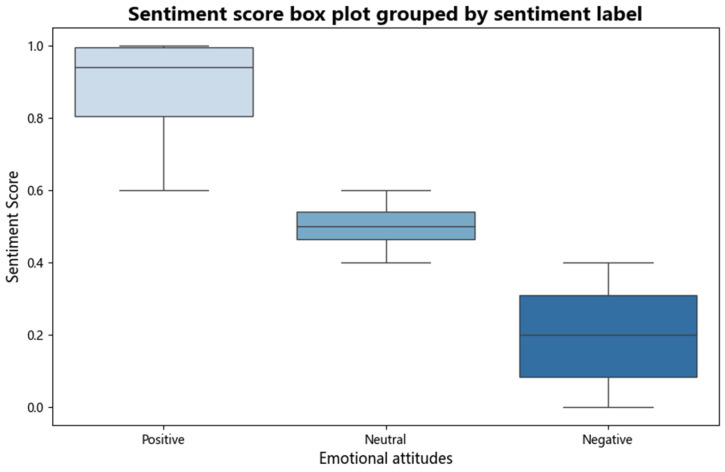
Sentimental score box plot.

By analyzing the sentimental score box map, the public’s attitude towards gamified education and its sentimental distribution can be evaluated. The box chart of Figure 6 shows that the positive sentiment score is concentrated between 0.8 and 1.0, with a median higher than 0.9, indicating that most comments have a positive attitude towards gaming education, believing that it can effectively improve learning experience and interest. The neutral sentiment score is around 0.5, which shows that the comments on gamified education are relatively neutral, with both recognition and reservations. Negative sentiment scores are concentrated between 0.1 and 0.3, and the boxplot is long, indicating that a small number of comments have negative views on gamified education, and may think that its effects are limited or there are unsuitable scenarios. Overall, the public’s attitude towards gamified education is mainly positive, neutral sentiments are moderate, while negative sentiments fluctuate greatly, which shows that they have a high acceptance of this educational method, but there are also certain uncertainties and doubts.

#### 4.1.3. Sentimental Attitude Analysis

**Figure 7:**
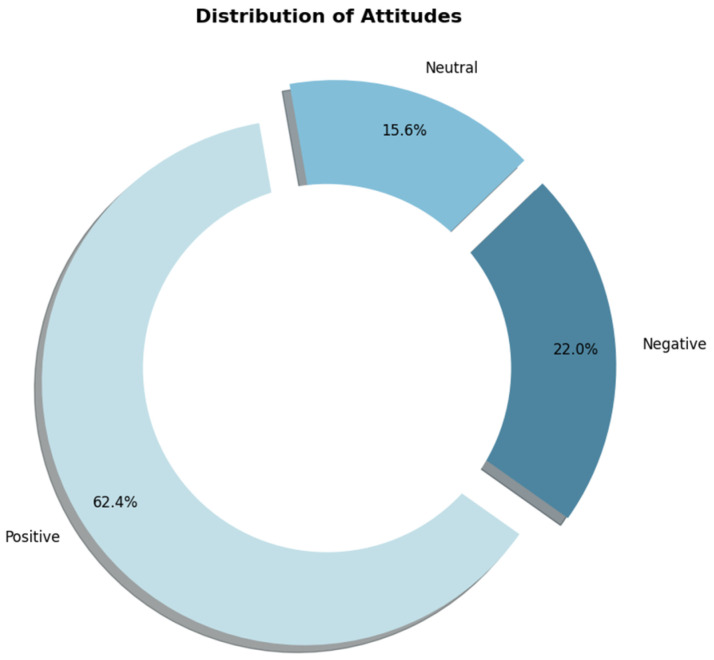
Sentimental attitude pie chart.

According to statistical analysis of the data, the public’s attitude towards gamified education is generally positive. As shown in Figure 7, 62.4% of commenters hold a “Positive” attitude, indicating that most people have a positive and positive view of gamified education, believing that its application in education can bring positive effects. Negative sentiments account for 22.0%, which shows that although most commenters have positive views, some people still object to gamified education, which may be due to doubts about its effect or applicable scenarios. In addition, 15.6% of commenters showed “Neutral” sentiments, did not express their sentimental tendencies clearly, and may be optimistic about gamified education. Overall, the public’s attitude towards gamified education is mainly positive and negative and neutral sentiments, which shows that gamified education is highly accepted by the public, but there are still some different views.

### 4.2. Potential Topic Analysis

**Figure 8:**
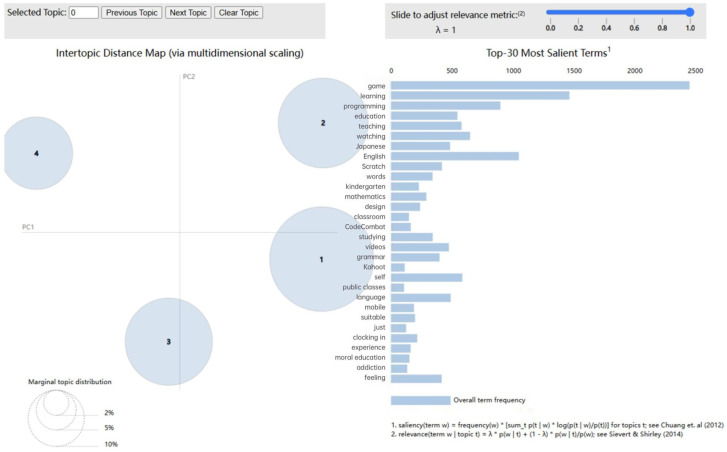
LDA visualized diagram of topic modeling(Chuang et al., 2012; Sievert & Shirley, 2014).

After repeated training and visualization of the generated topics using the LDAvis tool to provide a multidimensional model interpretation, Figure 8 shows that the four topics are clearly separated in the two-dimensional space, with no overlapping or intersecting areas. This demonstrates good discriminability and uniqueness, leading to the identification of the following four topics.

Topic 1: The Application of contextual strategies in language learning. It mainly discusses the auxiliary role of contextualized tools such as games and software in linguistic learning, such as “game”, “english”, “learn”, “japanese” and “grammar”. Situational strategies enhance the fun and interactivity of language learning by providing a real or close to real language environment, help learners effectively improve their vocabulary, grammar and comprehensive abilities, cultivate practical communication skills, and promote personalized and independent learning.

Topic 2: Autonomous Exploration and Active Participation in Gamified Learning. This topic focuses on independent learning through videos, application software, etc., with keywords such as “CodeCombat”, “Mobile phone”, “Check in”, “Own” and “Watch”. In a gamified learning environment, learners actively participate in learning and practice with the help of flexible rhythms and autonomously selected learning paths. Gamified learning stimulates learners’ intrinsic motivation by providing instant feedback and challenge mechanisms, so that they can independently adjust their learning strategies while enjoying a sense of accomplishment, thereby achieving a more efficient learning process.

Topic 3: Progressive Enhancement of Logical Thinking in Gamified Environments. This topic uses games as a learning medium and uses gamified teaching tools to help students gradually construct and master highly logical subject concepts in a relaxed and pleasant environment. Keywords such as “game”, “Scratch” (graphical programming enlightenment tool), “programming” and “math”, etc. Task design in a gamified environment follows the principle of progressive progress. As the task difficulty gradually increases, students need to continuously adjust their strategies, apply existing knowledge, and gradually improve their problem-solving ability. This gradual learning method helps students deepen their understanding of logical knowledge and cultivate and strengthen their logical thinking ability.

Topic 4: Teaching Innovation in Promoting Knowledge Construction and Deepening. Innovate teaching design through game formats to promote the construction and deepening of classroom knowledge, such as “design”, “teaching”, “game”, “curriculum” and “experience”. Gamified teaching methods use highly interactive task-driven and situational simulation to stimulate students’ learning motivation and promote the understanding and deepening of knowledge. In a relaxed learning environment, students use what they have learned in practice, think actively and interact, thereby gradually consolidating their knowledge and improving learning efficiency and interest. At the same time, this method can provide students with cognitive scaffolding, helping them internalize what they have learned in the process of gradual improvement and form a more stable knowledge system.

In summary, this study reveals individual sentimental differences across multiple themes through the complementary use of Method 1 and Method 2, while ensuring the clarity and robustness of the overall trend analysis. The results indicate that, overall, comments related to gamified education are predominantly positive, especially with high recognition in areas such as logical thinking training and teaching innovation. However, a certain proportion of neutral and negative feedback still exists within the themes of contextual strategies and self-directed exploration, primarily stemming from insufficient learning experiences or expectation gaps. This suggests that gamified education generally holds positive value, but certain aspects of the practice still need optimization to enhance learner engagement and satisfaction.

### 4.3. Topic Sentiment Analysis

**Figure 9:**
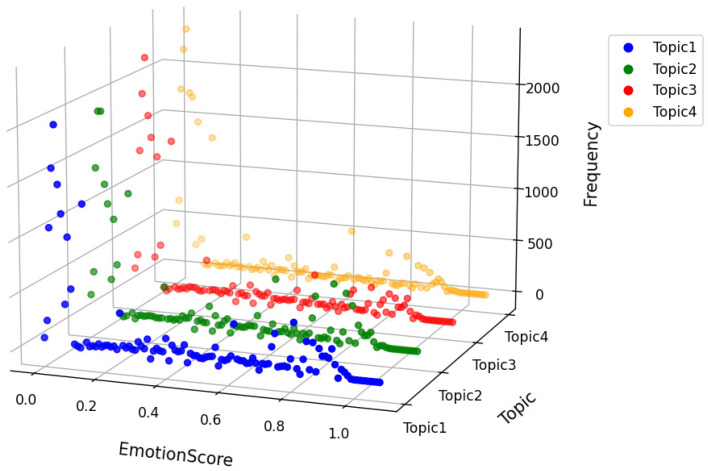
Sentiment chart under multidimensional topics.

First, a preliminary sentiment analysis was conducted on all comments based on the four topics, as shown in Figure 9. The sentiment expression of each comment varies across the four topics. Although each comment may focus on a particular topic, it can still exhibit varying degrees of sentiment inclination across all topics. For example, the comment “This game might be a bit boring for those who have learned programming, but it’s quite good for teaching purposes” has a relatively low sentiment score in Topic 1 and Topic 2, with scores of 0.08, respectively, indicating that the sentiment towards contextual strategy application and autonomous exploration is relatively neutral and weakly correlated. However, in Topic 3, the score is 0.27, reflecting a more negative view of gamified education in terms of cognitive training, with a moderate correlation. In contrast, the score for Topic 4 is 0.74, indicating that the reviewer has a positive sentiment towards teaching innovation and knowledge construction, with a strong correlation.

From the overall results, comments with scores near 0 are more prominent. The main reason for this is that these comments show no clear sentiment inclination toward the relevant topics, resulting in low correlation and an overall score close to 0. Meanwhile, the study found fluctuations in the scores of all topics around 0.8, suggesting a considerable number of comments with a positive attitude toward gamified education, and an overall trend of positive sentiment. However, some reviewers also expressed negative or neutral sentiments toward certain specific topics. This may be due to some reviewers’ insufficient experience with or unmet expectations regarding gamified education. In the future, educators should optimize relevant features and services based on these findings to improve the overall user experience.

**Figure 10:**
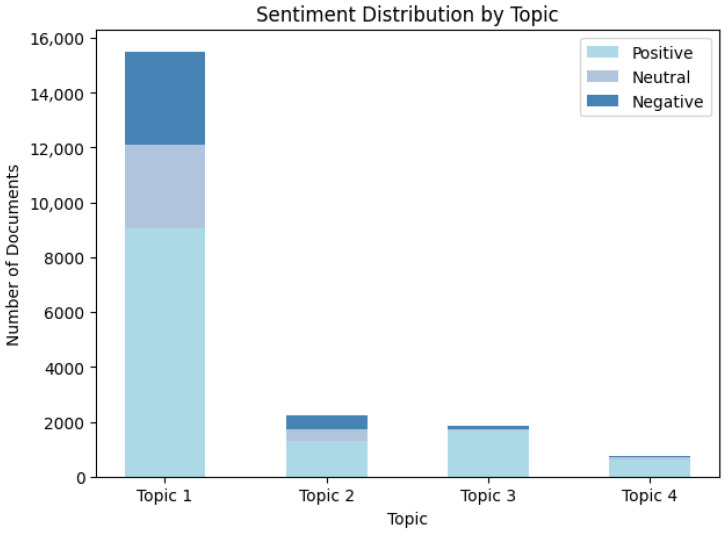
The sentiment map under the most relevant topic.

For a more in-depth analysis, each comment was categorized based on topic matching using sentiment vocabulary derived from the LDA model. This approach helps to more accurately associate comments with the sentiment of the corresponding topic, thereby further revealing the sentimental tendencies and focal points of commenters under different topics. Figure 10 shows the number of positive, negative, and neutral sentiment comments for each topic. The sentiment categories for each topic are distinguished by different colors in the chart, aiding analysts in better understanding the sentimental tendencies of comments under each topic.

Topic 1: The Application of Contextual Strategies in Language Learning. The total number of comments related to this topic is 15,501, with 9062 positive comments, accounting for approximately 58.5%; 3059 neutral comments, accounting for approximately 19.7%; and 3380 negative comments, accounting for approximately 21.8%. In the process of language learning, the proportion of positive sentiments is relatively high, indicating that learners generally recognize the effectiveness of using tools to enhance language learning. However, the presence of negative sentiments may be related to challenges such as learning difficulty, slow progress, or issues with tool usage.

Topic 2: Autonomous Exploration and Active Participation in Gamified Learning. The total number of comments related to this topic is 2215, with 1283 positive comments, accounting for approximately 58%; 423 neutral comments, accounting for approximately 19%; and 509 negative comments, accounting for approximately 23%. The high proportion of positive sentiments indicates that the self-directed learning approach has gained some recognition, but there is still considerable negative feedback, which may stem from a lack of interaction or immediate feedback during the self-learning process.

Topic 3: Progressive Enhancement of Logical Thinking in Gamified Environments. The total number of comments related to this topic is 1834, with 1667 positive comments, accounting for approximately 90.9%; 79 neutral comments, accounting for approximately 4.3%; and 88 negative comments, accounting for approximately 4.8%. The very high proportion of positive sentiments shows that gamified learning approaches are widely welcomed, especially in the fields of education and programming. However, a few comments express doubts regarding technical difficulty or learning outcomes.

Topic 4: Teaching Innovation in Promoting Knowledge Construction and Deepening. The total number of comments related to this topic is 743, with 634 positive comments, accounting for approximately 85.3%; 75 neutral comments, accounting for approximately 10.1%; and 34 negative comments, accounting for approximately 4.6%. This topic also shows a high proportion of positive sentiments, indicating that participants are generally favorable towards interactive design in teaching integration. The relatively small number of negative sentiments is primarily related to minor dissatisfaction with the use of tools or platforms.

The sentimental distribution of the four topics shows that positive sentiments dominate, especially in Topics 3 and 4, but the number of related comments is small, which may be because there are fewer people participating in game design, and due to the limitations of professional background, professionals with relevant experience are relatively scarce, so the number of comments is relatively low. Despite the dominance of positive sentiments, negative sentiments still exist in various topics, especially in topics one and two. Overall, although most feedback is positive, it also reminds us to focus on user experience and solve possible learning difficulties when designing learning tools and methods.

In summary, this study reveals individual sentimental differences across multiple themes through the complementary use of Method 1 and Method 2, while ensuring the clarity and robustness of the overall trend analysis. The results indicate that, overall, comments related to gamified education are predominantly positive, especially with high recognition in areas such as logical thinking training and teaching innovation. However, a certain proportion of neutral and negative feedback still exists within the themes of contextual strategies and self-directed exploration, primarily stemming from insufficient learning experiences or expectation gaps. This suggests that gamified education generally holds positive value, but certain aspects of the practice still need optimization to enhance learner engagement and satisfaction.

## 5. Discussion and Contribution

**Figure 11:**
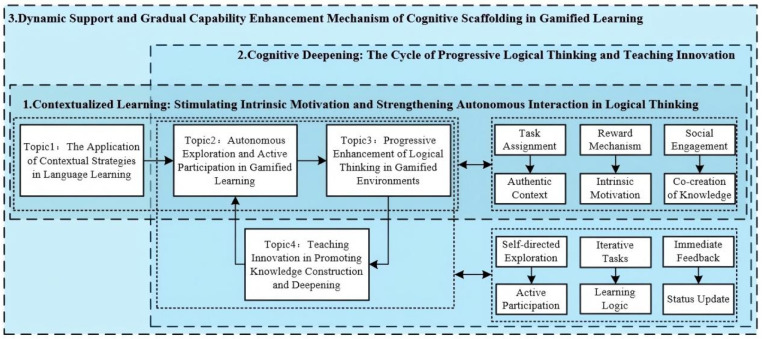
“Contextualization–Procedural Logic–Active Construction” Discussion Diagram.

This study has sentiment analysis and LDA theme modeling of gamified education related comments, and has in-depth discussion on the application of gamified education. The research results show that gamified education has shown positive potential in improving learning experience and effectiveness (Putz et al., 2020), but still faces some challenges (Alzahrani & Alhalafawy, 2022). Based on these findings, the following is a discussion of this study and related discussion figure (Figure 11):

### 5.1. Discussion of Sentimen Based on Topic Modeling

#### 5.1.1. Contextualized Learning: Stimulating Intrinsic Motivation and Strengthening Autonomous Interaction in Logical Thinking

Learners acquire practical skills and knowledge through interaction with their surrounding environment. This process is closely related to Situated Learning Theory (Marougkas et al., 2023), which emphasizes that learners gradually understand and master knowledge by participating in real-world social activities and interacting with their environment. Gamified learning, through design elements such as task assignment, reward mechanisms, and social engagement, aligns with principles like authentic context, intrinsic motivation, and co-creation of knowledge, thereby enhancing the contextualization of the learning process. It enables active exploration and participation in a relaxed and enjoyable setting (Azadnia, 2024). Through the design of authentic tasks, the learning content becomes relevant to the real-life needs of learners, thereby increasing the authenticity and perceived value of learning. The reward mechanism not only provides external incentives but also fosters the activation of intrinsic motivation through positive feedback (Zainuddin et al., 2020b). In the process of social engagement, individuals not only absorb knowledge but also negotiate meaning through dialogue, debate, and collaboration with others (Daghestani et al., 2020). Under the interwoven mechanisms, learners are able to explore and engage proactively in a pleasurable environment (Azadnia, 2024), continuously adjusting strategies and strengthening logical thinking through escalating challenges (Benton et al., 2021). However, in real-world scenarios, the use of related learning tools faces challenges such as technical issues, operational difficulties, or slow progress (Saleem et al., 2022), which can, in turn, affect the learners’ motivation and cause sentimental distress among the public (Eberle & Hobrecht, 2021; Meşe & Sevilen, 2021).

#### 5.1.2. Cognitive Deepening: The Cycle of Progressive Logical Thinking and Teaching Innovation

The process of cognitive deepening can be aligned with the “procedural logic” construct of the cognitive scaffolding model. In this process, learning is not simply the accumulation of knowledge, but rather the continuous deepening of thought and enhancement of abilities through progression and innovation. Initially, self-directed exploration and active participation serve as the starting phase of learning. Learners, based on their individual needs, actively choose learning tasks (Rodrigues et al., 2022), and through exploration and interaction, they construct an initial understanding of knowledge, thus engaging actively in the process of co-creation of knowledge and continuously forming their own learning pathways (Zourmpakis et al., 2023). Subsequently, iterative tasks in the game design progressively advance the learning logic. Each learning task is built upon the knowledge from the previous phase, and this gradual learning approach not only helps learners deepen their understanding of knowledge (O’Brien & Battista, 2020) but also promotes the enhancement of logical thinking and problem-solving abilities. This design, akin to “procedural logic”, is both a manifestation of cognitive deepening and a reflection of the cyclical mechanism of teaching innovation. Additionally, immediate feedback represents a status update. When learners complete tasks and receive feedback (Wulantari et al., 2023), game-based innovations guide reflection on the learning process and transition to the next phase, stimulating learners to apply and internalize higher-level knowledge within the knowledge system (Lombardi et al., 2021). Ultimately, this creates a closed-loop ascending mechanism, where learners, driven by each task, gradually progress towards more self-directed and deeper cognitive levels, forming a continuous self-improvement learning process.

#### 5.1.3. Dynamic Support and Gradual Capability Enhancement Mechanism of Cognitive Scaffolding in Gamified Education

In gamified learning, Cognitive Scaffolding corresponds to the “active meaning-making” dimension of the cognitive scaffolding model. As an external support mechanism, Cognitive Scaffolding plays a crucial role in gamified learning. It provides learners with flexible and dynamic support during the learning process, enabling them to gradually acquire the necessary skills while solving authentic problems (Doo et al., 2020). Within authentic contexts of situated learning, the design of iterative tasks effectively reflects the principle of progressive skill differentiation. In this process, Cognitive Scaffolding facilitates learners’ cognitive growth and problem-solving strategies by gradually increasing Task assignment difficulty and reinforcing the feedback mechanism (Y. Lee et al., 2023). The feedback mechanism embodies the core function of dynamic support (Lave & Wenger, 1991): through immediate feedback, learners receive concrete, directional guidance that prompts them to continuously adjust strategies during problem-solving. This not only optimizes their learning logic but also advances cognitive development (Al Mamun & Lawrie, 2024). Furthermore, reward mechanisms and achievement systems in gamified design effectively enhance learners’ self-efficacy, reinforcing intrinsic motivation through a positive psychological cycle that sustains long-term engagement (Yu et al., 2024). Research indicates that learners generally hold positive attitudes toward Cognitive Scaffolding in gamification. They recognize that its progressive support and feedback mechanisms not only improve cognitive competence but also foster enduring motivation for continuous learning (Lin et al., 2022).

### 5.2. Research Contributions and Inspiration

Gamified education will show broad application prospects in the future development of education, which will not only provide learners with a richer learning experience, but will also have a profound impact on various educational practices.

#### 5.2.1. The Main Contributions of This Study Are as Follows

**A. Expanding the Application of Situated Learning in the Cognitive Scaffolding Model.** Situated Learning Theory advocates that learning should be closely linked to real-world contexts, embedding learning content into social interactions and practical applications. In gamified education, the potential pathway for situated learning involves placing learners in highly interactive contexts, enhancing their learning motivation and sense of participation, and stimulating active exploration in learning. Next, in logical thinking training, learners are enabled to closely combine situational and logical aspects, embedding rational awareness into their cognitive training. Finally, in the cognitive scaffolding model, the innovation of teaching and the construction and deep interpretation of knowledge are realized. This cognitive development framework formed by topic modeling further extends the scope and explanatory framework of Situated Learning Theory and aligns with the development of the cognitive scaffolding model.

**B. Combining LDA Topic Modeling and Sentiment Analysis for Multi-dimensional Sentiment Measurement.** This study combines LDA topic modeling with sentiment analysis to achieve precise modeling and multi-dimensional measurement on the topic dimension. Using the first method, the topic-word distribution matrix output by the LDA model links sentiment scores of reviews with specific topics, revealing sentimental differences within the same review across different topics, thus supporting deeper sentiment attitude analysis. The second method constructs semantic relationships between reviews and topic keywords, applying the similarity maximization principle for topic classification, which further enhances the topic relevance and interpretability of sentiment analysis. This dual analysis strategy combining LDA and sentiment analysis allows for a comprehensive evaluation of user sentimental attitudes from both a holistic and segmented perspective, thereby providing more comprehensive, accurate, and targeted research conclusions.

#### 5.2.2. Practical Recommendations

**A. Integrating Usability Heuristic Principles with Gamification Design Frameworks.** The design of learning tools should adhere to usability heuristic principles, ensuring that operations are simple, feedback is clear, and navigation is consistent, in order to prevent complex interfaces from undermining intrinsic motivation (Xiao, 2022). In addition, gamification design frameworks, such as Octalysis and MDA (Ivanova & Georgiev, 2019), should be incorporated, utilizing elements such as task gradation, immediate feedback, reward mechanisms, and social engagement to enhance immersion (Dichev et al., 2015). The design should strike a balance between functional usability and motivational drive, avoiding excessive complexity. For example, improvements in interface design (Triantafyllou & Georgiadis, 2022), reduction in technical barriers (Sousa et al., 2023), and enhancement of operability and platform stability (K. Lee, 2023) are necessary considerations.

**B. Scaffolding in Gamified Learning: Emphasizing Dynamism and Personalization.** Scaffolding in gamified learning emphasizes self-directed learning and personalization. To enhance its effectiveness, iterative A/B testing can be used to compare the impact of different scaffolding designs (such as task difficulty, feedback frequency, etc.) on learners, thereby optimizing learning paths and personalized scaffolding designs (Shin et al., 2020). By comparing different versions of designs, such as incremental task difficulty, feedback frequency, and reward types, the influence of these factors on learning motivation, cognitive load, and learning outcomes can be analyzed, and the scaffolding mechanism can be refined accordingly (Sun et al., 2018). A/B testing should be embedded in a long-term iterative process (Bojinov & Gupta, 2022), forming a “trial-feedback-optimization” feedback loop that ensures scaffolding mechanisms adjust as learners’ cognitive abilities develop, ultimately improving learning outcomes.

**C. Integrating Situated Learning with Cognitive Scaffolding Design.** The integration of situated learning with Cognitive Scaffolding can enhance learning outcomes. First, tasks should be connected to authentic contexts or simulated real-world scenarios (Alvear et al., 2021; Ivanjko et al., 2024). Second, Cognitive Scaffolding needs to gradually increase task difficulty while providing timely feedback and rewards to help learners improve their abilities. Finally, sentimental support plays a crucial role, where social engagement through cooperation or competition can alleviate sentimental distress (Castañer & Oliveira, 2020). For instance, in medical education, a simulation game designed for emergency care could help learners progress from simple to complex cases, gradually improving their cognitive skills, while team-based cooperation fosters sentimental engagement (Elendu et al., 2024).

## 6. Conclusions

This study, based on 20,293 user comments from the Bilibili platform, combines sentiment analysis and LDA topic modeling methods to systematically reveal the multi-dimensional effects of gamified education in enhancing learner sentimental engagement, motivating learning, and promoting knowledge construction. The study found that positive emotions dominate in user feedback, especially in areas such as language learning, autonomous exploration, logical thinking training, and teaching innovation, which validates the adaptability and effectiveness of Situated Learning Theory in gamified design. However, some comments still reflect issues such as complex operations, lack of interactivity, and feedback delays, suggesting that there is room for improvement in the user experience of current gamified education tools.

Through the identification and analysis of four themes, the study further developed a cognitive scaffolding model integrating “situatedness—program logic—active construction” to provide theoretical support for understanding the cognitive and sentimental interaction mechanisms in gamified education. This model not only expands the application boundaries of Situated Learning Theory in digital educational environments but also provides a practical cognitive structure framework for future gamified teaching design.

Although this study demonstrates certain innovations in methodological integration and theoretical construction, its data sources have certain limitations. The data currently used mainly focuses on online text feedback from users in mainland China, and this singular sample structure may limit the applicability of the research findings in cross-cultural contexts. Given the significant differences in sentimental expression, social interaction preferences, and learning motivation across various cultural backgrounds, the conclusions of this study may exhibit varying applicability or effects in other cultural contexts. Therefore, future research should appropriately expand the sample source to include users from different countries and cultural backgrounds, and explicitly control for cultural factors in the research design (Spector et al., 2015). Additionally, there is room for improvement in the research methodology. Solely relying on SnowNLP for sentiment analysis has certain limitations. While this tool performs well in sentiment analysis of Chinese text, its algorithm depends on pre-trained dictionaries and corpora, which may lack the adaptability needed for domain-specific terminology, contextual relevance, and the handling of emerging internet slang, regional dialects, and sentimental expressions in cross-cultural settings. This could lead to biases in sentiment classification. Future studies could combine facial expression recognition and micro-expression capture technologies to assist in validating the sentimental orientation of the text (K. Patel et al., 2020; Zhu et al., 2022). Additionally, methods such as in-depth interviews and contextual playback could be employed to gather learners’ sentimental experiences during specific learning tasks, thereby obtaining richer sentimental–behavioral data (Deterding & Waters, 2021; Sasangohar et al., 2021). This multi-method approach would enable a more comprehensive, multimodal understanding of learners’ sentimental attitudes.

In summary, gamified education, as a teaching model integrating technology and cognitive strategies, shows great potential in enhancing learning experiences, boosting motivation, and optimizing sentimental feedback. Educators and platform developers should continuously optimize gamified design logic and interaction mechanisms based on learners’ real needs and sentimental responses, promoting deeper knowledge construction and educational equity in diverse, highly interactive learning environments.

## Figures and Tables

**Table 1:** SnowNLP performance test.

Group	Accuracy	Precision	Recall	F1 Score
1	0.71	0.68	0.74	0.71
2	0.73	0.70	0.75	0.73
3	0.75	0.73	0.77	0.75
4	0.72	0.69	0.72	0.71
5	0.73	0.70	0.75	0.73

**Table 2:** List of keywords.

Num	Word	Frequency
1	game	1956
2	learn	1442
3	English	1116
4	programme	881
5	video	649
6	software	568
7	oneself	557
8	education	471
9	teach school	465
10	Japanese	459

## Data Availability

The data used in this study is available upon request. Please contact the corresponding author via email for access.
